# Interpreting the New Competency-Based Medical Education Curriculum of Biochemistry for Undergraduate Medical Students in India: A Narrative Review

**DOI:** 10.7759/cureus.108578

**Published:** 2026-05-10

**Authors:** Nhetan Navin Acharya, Koushik Biswas, Pratibha Gavel

**Affiliations:** 1 Biochemistry, All India Institute of Medical Sciences, Raebareli, IND; 2 Biochemistry, Homi Bhabha Cancer Hospital and Mahamana Pandit Madan Mohan Malaviya Cancer Centre, Varanasi, IND

**Keywords:** biochemistry curriculum, bloom’s taxonomy, competency-based medical education, competency-based undergraduate curriculum, dreyfus model, indian medical graduate, integrated teaching, medical student assessment, millers pyramid, undergraduate and graduate medical education

## Abstract

The Graduate Medical Education Regulations were amended in 2018 to implement the new competency-based medical education curriculum for undergraduate medical students in India from the 2019 admission batch onwards. In this review, we have analyzed the changes introduced in the new curriculum in the discipline of Biochemistry in terms of competencies, domains, levels of competency, teaching-learning methods, assessment methods, certifications, and integrations. Thereafter, we have synthesized and presented our analysis as a narrative review. In this new curriculum, competencies are presented in a tabular form. There are 89 competencies in Biochemistry, including 24 practical competencies. This Biochemistry curriculum covers 77 knowledge and 12 skills competencies. The attitude and communication aspects are taught separately to Phase I undergraduate medical students in Attitude, Ethics, and Communication modules. Teaching-learning methods in Biochemistry include lectures, small-group discussions, bedside clinics, practicals, demonstration-observation-assistance-performance, and skills lab sessions. For Biochemistry, 82 hours of large-group teaching, 157 hours of small-group teaching/practical/tutorial/seminar, and 10 hours of self-directed learning are recommended. The lower tiers of the Miller’s pyramid of clinical competence, “knows” and “knows how,” are assessed by the written theory examination, while the “shows how” level is assessed by objective structured clinical examinations and direct observation during practical procedure. Eight Physiology competencies are horizontally integrated with the Biochemistry curriculum. 8 Pathology, 4 Dermatology, 1 Ophthalmology, 11 General Medicine, 41 Pediatrics, and 4 General Surgery competencies are vertically integrated with the Biochemistry curriculum. Integrated teaching provides early clinical exposure and helps undergraduate medical students gain a blended concept by linking the topics from the viewpoint of different disciplines.

## Introduction and background

Medical education has long been structured around discipline-based teaching and summative examinations, with success often measured by performance in high-stakes entrance and exit assessments. While this model has ensured theoretical grounding, it has increasingly been perceived as inadequate in producing graduates who are clinically competent, professionally responsible, and responsive to societal needs [1]. Concerns expressed by both graduates and faculty regarding “hyposkillia,” the gap between acquired knowledge and practical ability, have highlighted the limitations of an education system that places disproportionate emphasis on examination performance rather than clinical competence [2]. The growing dominance of competitive examinations, such as postgraduate entrance tests, has further reinforced a knowledge-centric learning culture at the expense of skill acquisition, communication, and ethical practice [3]. Simultaneously, changing societal expectations, an increase in transparency in healthcare delivery, and heightened accountability of medical professionals have necessitated a reorientation of undergraduate medical training [4]. Competency is defined as “the ability to do something successfully and efficiently,” while competency-based medical education (CBME) is “an approach to ensure that the graduates develop the competencies required to fulfill the patients’ needs in society” [5].

The origin of the competency approach came from parallel progress in vocational training in multiple countries such as Australia, New Zealand, the United Kingdom (UK), and the United States of America. The country's policymakers believed that healthcare workers needed to become more competitive in the global market, which became a major driving force behind initiating this competency approach in training. The national vocational credentials in the UK were created as a set of standards, each of which is divided into components that can be used to evaluate performance in the workplace. Since then, this method has been used for instruction in a variety of professions, especially technical and vocational ones [6].

In this context, the concept of competency has emerged as a unifying framework that emphasizes what a medical graduate is able to do in real-life professional settings. CBME builds upon this premise by restructuring curricula around clearly defined outcomes encompassing knowledge, skills, attitudes, and communication [5].

Competency in medical education is a multidimensional construct that extends beyond the mere acquisition of factual knowledge and technical proficiency. Within the framework of CBME, competencies are conceptualized as an integrated combination of abilities that enable a medical graduate to function effectively in real-world healthcare settings. These components can be broadly understood across three interrelated dimensions: the ability to perform appropriate clinical tasks, the manner in which medical practice is approached, and the development of the doctor as a professional individual [7]. The first component focuses on doing the right thing, which refers to the demonstrable technical and clinical competencies expected of a medical graduate. This includes the ability to perform clinical skills and practical procedures, appropriately select and interpret investigations, manage patients effectively, promote health and disease prevention, and communicate relevant information accurately [7,8]. The second component emphasizes how the doctor approaches medical practice, integrating cognitive and affective elements of competence. This dimension includes scientific understanding, ethical attitudes, sound clinical decision-making, and the ability to function effectively within a healthcare team. It recognizes that competent performance is not solely defined by task completion but also by the quality of reasoning, professionalism, and collaboration underlying clinical actions [7,9]. The third component highlights the right person doing it, underscoring the importance of professional identity formation. This dimension encompasses personal development, accountability, self-reflection, and a commitment to lifelong learning. CBME acknowledges that medical education must cultivate not only competent practitioners but also responsible professionals who can adapt to evolving healthcare demands. Embedding this component within undergraduate training encourages early development of professionalism, integrity, and reflective practice, which are essential attributes of a competent medical graduate [7].

The design of the CBME model follows a structured, cyclical, and outcome-driven approach that ensures alignment between educational intent and learner performance. The process begins with the identification of clearly defined desired outcomes that represent the abilities a medical graduate is expected to demonstrate upon completion of his/her training. These outcomes are then translated into specific competencies, with each competency assigned a defined level of performance based on established educational frameworks. Once competencies are articulated, appropriate teaching-learning methods are selected to facilitate their acquisition, ensuring alignment with the cognitive, psychomotor, affective, and communication domains of learning [10].

The CBME model emphasizes the development of a robust assessment framework that incorporates multiple tools aligned with the level of competency expected, thereby enabling meaningful evaluation of learner progression. Central to this model is the provision of timely, structured feedback, which supports reflective learning and continuous improvement rather than mere judgment of performance. Importantly, CBME is not a static curriculum; it requires continuous program evaluation and refinement, ensuring that teaching strategies, assessment methods, and learning experiences remain responsive to learner needs and evolving healthcare demands. This iterative cycle is sustained until the desired competency outcomes are demonstrably achieved, reinforcing CBME as a dynamic, learner-centered educational model [10,11].

## Review

Methods

We searched the traditional undergraduate medical curriculum in India, which was published in 1997 [12]. Next, we searched for the competency-based undergraduate medical curriculum in India, which was published in 2018 [13]. We analyzed the changes in this new curriculum in the discipline of Biochemistry in terms of the competencies, domains, levels of competency, teaching-learning methods, assessment methods, certifications, and integrations. Given the nature of the available data (policy documents and curricular frameworks), a quantitative meta-analysis was not feasible; therefore, a narrative synthesis was undertaken. All figures were drawn in Microsoft PowerPoint (Microsoft Corp., Redmond, WA, USA) and then converted to JPG format by CloudConvert (Lunaweb GmbH, Munich, Germany) (https://cloudconvert.com/ppt-to-jpg).

Findings

Role of Indian Medical Graduate

The Graduate Medical Education Regulation (GMER) implemented in India in 1997 emphasized that Indian Medical Graduates (IMGs) must be able to effectively function as a clinician, leader and member of the healthcare team, communicator, lifelong learner, and professional. As a clinician, an IMG should be able to provide preventive, curative, and palliative care to patients. As the leader of the healthcare team, an IMG must be able to collect data from the hospital/community, analyze it, and communicate the findings to the higher authority (the health department in most cases). An IMG should empathetically communicate with patients, patient families, community residents, and their own colleagues. To be a lifelong learner, an IMG can participate in conferences/workshops/continuous medical education programs/government-approved trainings, and continuously improve their skills and knowledge. An IMG should be a professional, practice ethically, be responsive to patients, and remain committed to excellence. In India, CBME was introduced for the training of medical undergraduates from the 2019 academic session onwards [14]. 

*Traditional vs. CBME System* 

The recent adoption of CBME in the medical curriculum in India for undergraduates represents a fundamental shift in educational philosophy [15]. In the Phase I subject, such as Biochemistry, aligning conceptual understanding with clinical application is of prime importance. Interpreting this curricular transformation through the perspectives of teachers and students is essential to acknowledge how CBME can move beyond policy intent to meaningful educational and clinically sound practice [10].

The traditional medical education system has largely been structured around time-based training, content delivery, and summative assessment, where progression is determined by the duration of study and performance in examinations rather than demonstrated ability. Within this framework, emphasis is placed predominantly on theoretical knowledge acquisition, often resulting in limited attention to practical skills, clinical reasoning, communication, and professional development. In contrast, CBME represents a fundamental shift toward outcome-oriented training, wherein the central focus is on how the learner is able to inculcate whatever has been acquired at the end of the educational process. CBME prioritizes the acquisition of clearly defined technical and non-technical competencies, including clinical skills, procedural proficiency, investigative reasoning, patient management, health promotion, communication, and information handling. Beyond task performance, the competency approach also emphasizes conduct in medicine, integrated scientific understanding with ethical attitudes, decision-making abilities, and teamwork [6,15]. The differences between the traditional system and CBME are presented in Table 1.

**Table 1:** Differences between the traditional and competency-based medical education system

Traditional system	Competency-based medical education
Curriculum	
Subject centered	Learner centered
Time based	Not time based
Assessment	
Summative	Formative
Written and viva	Assesses the defined desired competency
Little feedback	Feedback is a process of training
Teaching Learning Methods	
Focus on knowledge	Focus on attitude and skills
No focus on soft skills	Focus on soft skills

Furthermore, CBME recognizes the importance of professional identity formation, underscoring personal development and accountability as integral components of medical training. By emphasizing a learner-centered, performance-based framework, CBME seeks to bridge the gap between education and real-world clinical practice, thereby addressing longstanding concerns regarding graduate preparedness and professional competence [16].

Curriculum Structure: Understanding the Competency Table in the CBME Curriculum

The CBME Biochemistry curriculum is present in the UG curriculum Volume I, pages 119-135, published by the Medical Council of India in 2018 [13]. The curriculum has been presented in a table with 10 columns. The first column denotes the unique number of the competency. The second column denotes the description of the competency. The third column identifies the domain(s) addressed by the competency: knowledge, skill, attitude, or communication. The fourth column identifies the competency level on the basis of Miller’s pyramid: know (K), know how (KH), show (S), show how (SH), or perform (P). The fifth column denotes if the competency is core or desirable. The sixth and seventh columns mention the suggested teaching-learning method and the suggested assessment method for the competency, respectively. The eighth column denotes how many times a skill is required to be carried out independently. The ninth and tenth columns denote the possibility of vertical and horizontal integration, respectively, for a better understanding of the competency (Table 2) [13]. The competency table will help the teacher to frame the aims and objectives of the class during lesson planning.

**Table 2:** Explanation of different columns of the competency table in the competency-based medical education curriculum

Column of competency table	Denotes
1	Unique competency number
2	Description of competency
3	Learning domains addressed: knowledge (K), skill (S), attitude (A), communication (C)
4	Level of competency required based on Miller’s pyramid: know (K), know how (KH), show (S), show how (SH), perform (P)
5	Check if the competency is core or desirable
6	Suggested teaching-learning method
7	Suggested assessment method
8	Number of times a skill needs to be independently performed
9	Possibility of vertical integration (if any)
10	Possibility of vertical integration (if any)

Learning Domains

A committee led by Benjamin Bloom identified three domains of learning, such as cognitive, psychomotor, and affective, which broadly signify the knowledge (K), skill (S), and attitude (A) components of learning, respectively [17]. The communication (C) component was not a part of the traditional curriculum but has been introduced under the CBME curriculum. Communication is important for medical students for explaining patient risks to their relatives, breaking bad news, counseling cases of bereavement or accidents, informing the benefits and risks of a surgical procedure, and taking informed consent [18]. The CBME Biochemistry curriculum of India covers 77 knowledge (K) and 12 skills (S) competencies. Attitude and communication are taught separately to Phase I undergraduate medical students in the Attitude, Ethics, and Communication (AETCOM) modules [19]. The cognitive, psychomotor, and affective domains need to be achieved in parallel, not step-by-step [20]. Undergraduate medical students are taught five AETCOM modules over 34 hours during their Phase I (first year) [21]. An example of these domains in Biochemistry for undergraduate medical students is presented in Table 3.

**Table 3:** Domains of learning in the context of Biochemistry for Phase I undergraduate medical students

Domain	Learning example for undergraduate medical students
Cognitive (knowledge)	Knowing the diagnostic criteria of diabetes mellitus
Affective (attitude)	Counseling a newly diagnosed patient with diabetes in an empathetic manner
Psychomotor (skill)	Performing a capillary blood glucose test

Levels of Competence

**Figure 1:**
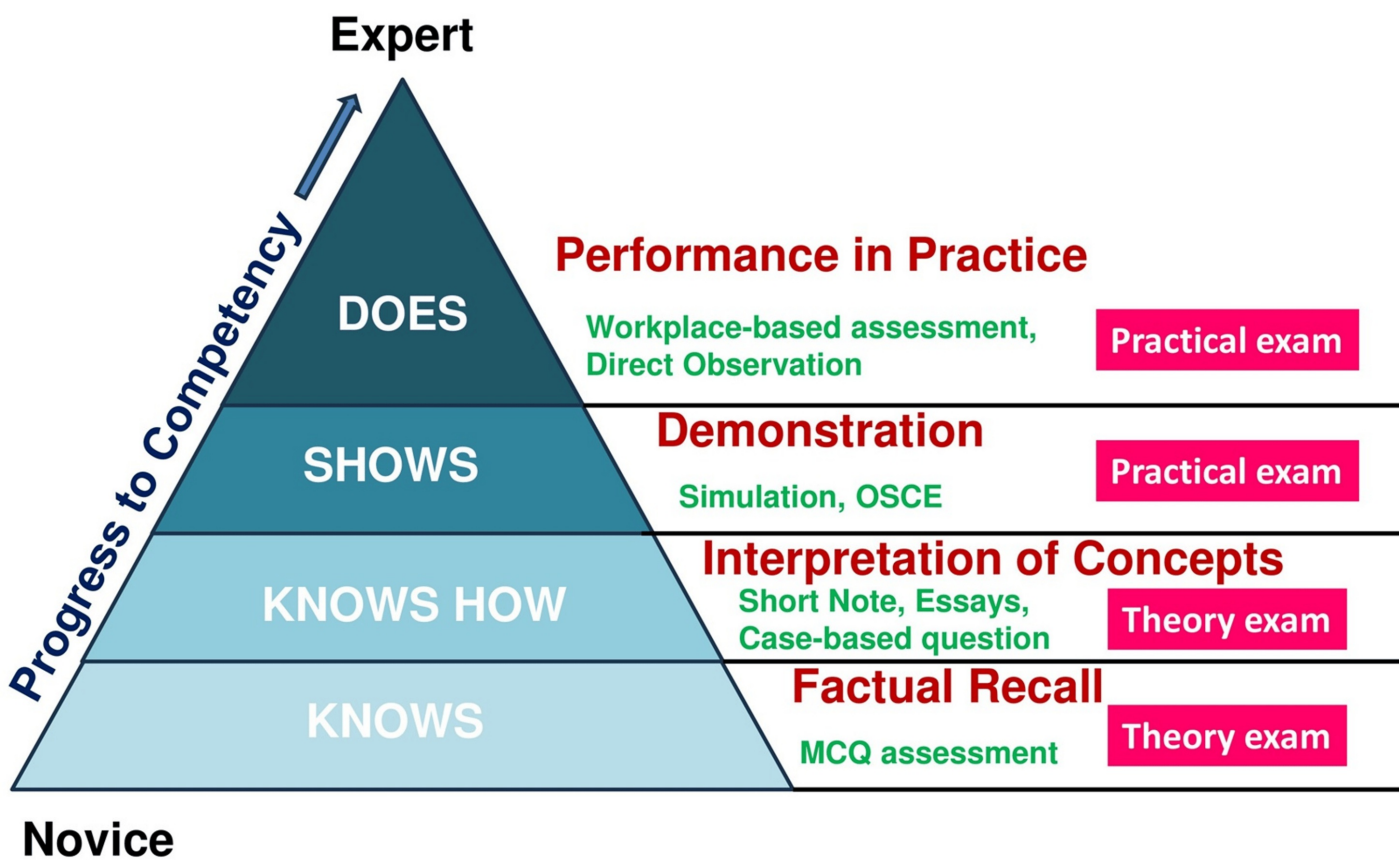
Miller’s pyramid of clinical competence in the context of undergraduate medical students in India MCQ: multiple choice question; OSCE, objective structured clinical examination. The concept of figure is attributed to Ramani and Leinster [23]. The figure has been modified and reproduced with permission from the author and Taylor & Francis. Images are created by authors using Microsoft PowerPoint (Microsoft® Corp., Redmond, WA).

The application of Miller’s pyramid of clinical competence within the framework of CBME, highlighting the progressive integration of knowledge, skills, and attitudes across different levels of learner performance, is presented in Figure 1. The lower tiers of the pyramid, “knows” and “knows how,” represent foundational cognitive processes, including factual recall and the application of knowledge, which are predominantly assessed through theory-based examinations such as multiple-choice questions (MCQs), written tests, and case-based questions. As learners progress upward, the “shows how” level emphasizes demonstration of competence in controlled or simulated settings, such as objective structured clinical examinations and practical demonstrations, bridging the gap between theoretical understanding and applied skills [22,23]. In the context of undergraduate Biochemistry, this model reinforces the CBME mandate to move beyond exclusive reliance on theory examinations and to incorporate structured practical assessments that evaluate laboratory skills, interpretation of investigations, and professional behavior. From the teachers’ perspective, the pyramid provides clarity in aligning assessment tools with expected competency levels, while for students, it offers transparency regarding progression from knowledge acquisition to real-world performance, thereby operationalizing the intent of CBME within foundational medical sciences.

Stages of Competence and Skill Acquisition: The Dreyfus Model

**Figure 2:**
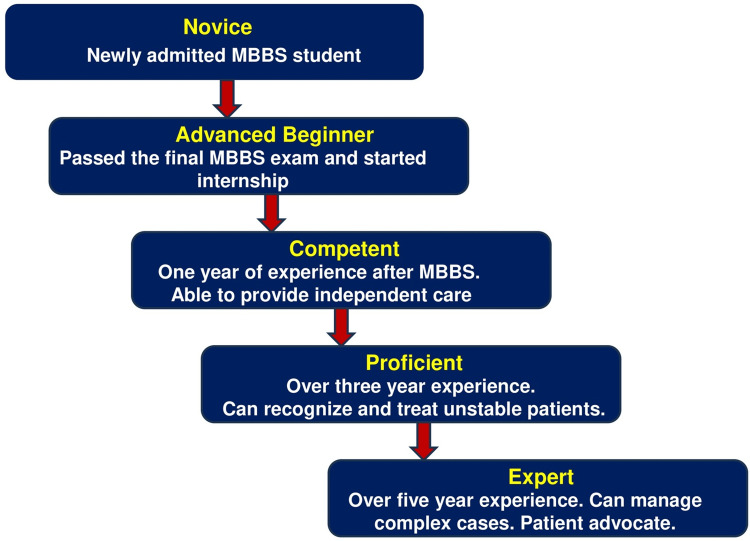
Stages of competence and skill acquisition MBBS, Bachelor of Medicine and Bachelor of Surgery. The concept of figure is attributed to Peña [24]. Non-commercial reproduction with proper citation is permitted by the journal. Images are created by authors using Microsoft PowerPoint (Microsoft® Corp., Redmond, WA).

Complementing Miller’s pyramid, the Dreyfus model of skill acquisition offers a developmental perspective on how learners progress through stages of competence over time. This model describes five stages -- novice, advanced beginner, competent, proficient, and expert -- each stage is represented by distinct patterns of learning, decision-making, and performance (Figure 2) [24]. At the novice stage, learners rely heavily on rules and guidelines, requiring close supervision and explicit instruction. In undergraduate Biochemistry, this corresponds to early exposure to laboratory techniques, reagent principles, and basic interpretation rules. During the period of the Bachelor of Medicine and Bachelor of Surgery (MBBS) course, a learner progresses from novice to advanced beginner.

Competency: Number and Types

The CBME Biochemistry curriculum for medical undergraduates in India has 89 competencies across 11 topics. The topic-wise number of competencies and the number of procedures that require certification for each competency are mentioned in Table 4. Of these 89 competencies, 88 (98.9%) are core competencies that a student must know. Only one (1.1 %) is a desirable competency.

**Table 4:** Topic-wise number of competencies in the competency-based medical education Biochemistry curriculum

Topic	Number of competencies	Number of procedures that require certification
Basic Chemistry	01	Nil
Enzyme	07	Nil
Chemistry and Metabolism of Carbohydrate	10	Nil
Chemistry and Metabolism of Lipids	07	Nil
Chemistry and Metabolism of Proteins	05	Nil
Metabolism and Homeostasis	15	Nil
Molecular Biology	07	Nil
Nutrition	05	Nil
Extracellular Matrix	03	Nil
Oncogenesis and Immunity	05	Nil
Biochemical Laboratory Tests	24	05
Total	89	05

Teaching-Learning Strategies

The CBME curriculum highlights various teaching-learning methods for teaching undergraduate medical students. These include lectures, small group discussions, bedside clinics, practicals, demonstration-observation-assistance-performance, and skills lab sessions. A notification issued by the National Medical Commission, India, in September 2024 recommended the following for Phase I Biochemistry: 82 hours of large group teaching, 157 hours of small group teaching/practical/ tutorial/seminar, and 10 hours of self-directed learning, making the total teaching-learning activity 249 hours [25].

Assessment Framework

Three types of assessment methods used during summative assessment of undergraduate medical students are written, viva voce, and skill assessment (practical/clinical examinations). As per GMER 2018, for Biochemistry assessment, there should be two theory papers (100 marks each) and a practical examination (including viva voce) of 100 marks. The theory question paper is required to be designed taking into consideration all levels of the knowledge domain (knowledge, comprehension, application, analysis, synthesis, evaluation) as mentioned in Bloom’s taxonomy of cognitive domains. Theory examination paper should consist of long answer questions (structured essay type), short answer questions, and objective-type questions (such as MCQs). Regulations highlight that the weightage of MCQs should not have more than 20% weightage in the theory examination [26,27]. Scenario-based MCQs have become popular in recent years, as they can evaluate a student's knowledge, critical thinking, and problem-solving abilities in real-world situations [28].

The practical (including viva voce) examination should include assessment of the psychomotor and affective domains. For Biochemistry, the practical assessments should be based on direct observation of students by examiners while they are doing a laboratory test. In Phase I subjects such as Biochemistry, objective structured practical examination, directly observed procedural skills, or a one-minute preceptor may also be used for student evaluation. GMER 2018 recommends using multiple examiners for student assessment to minimize subjectivity. This can also indirectly provide training to junior faculties and senior residents on different aspects of student assessment [26].

Practising a Skill Independently

**Figure 3:**
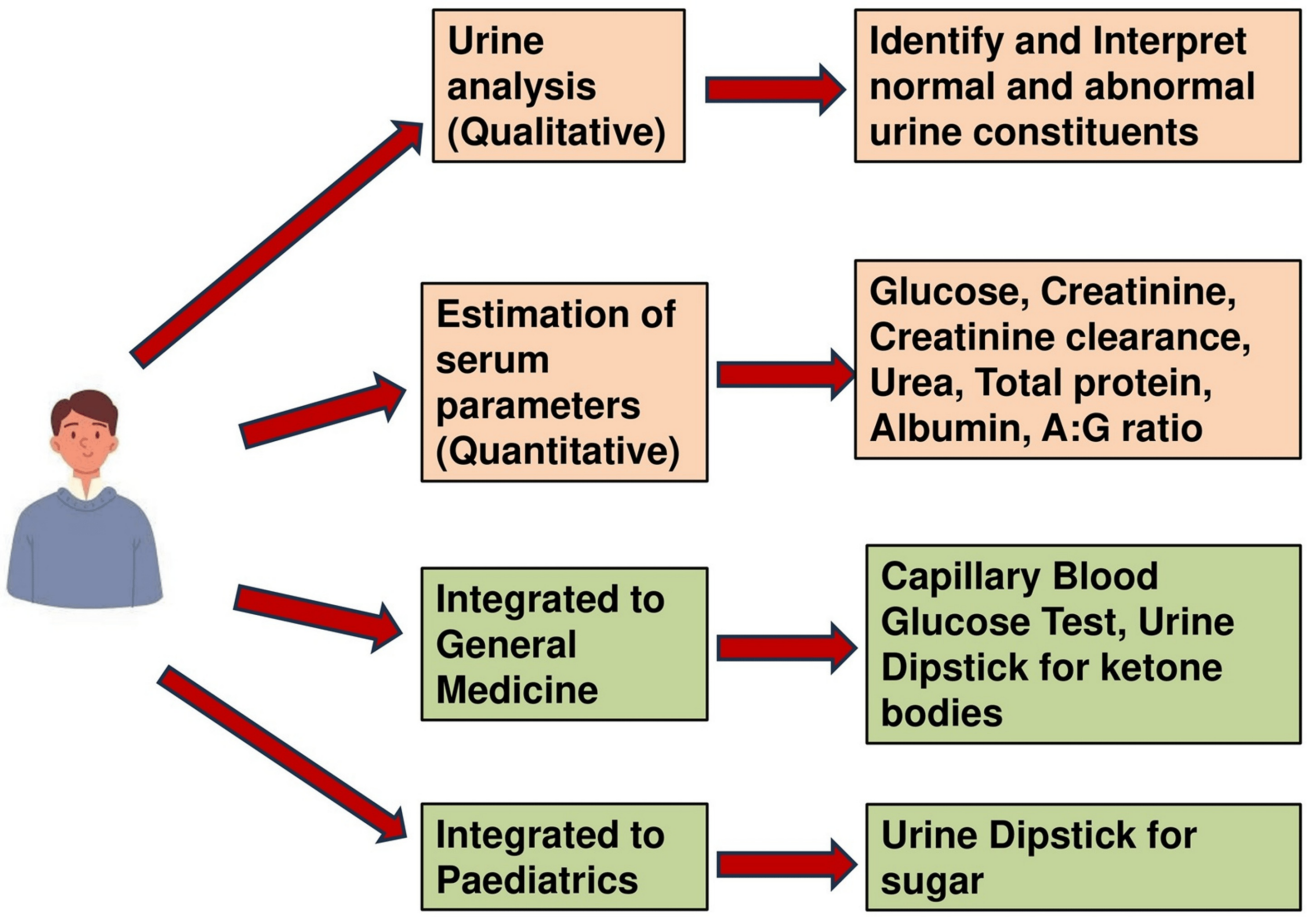
Practical skills in Biochemistry that require independent practice Skills mentioned in orange boxes are core Biochemistry practicals, while those mentioned in green boxes are integrated with other departments. Images are created by authors using Microsoft PowerPoint (Microsoft® Corp., Redmond, WA).

The CBME curriculum mentions that a few practical skills have to be performed independently, as shown in Figure 3. Each student is required to independently perform the Biochemistry practicals, viz., estimation of serum glucose, urea, creatinine, creatinine clearance, total protein, albumin, and albumin:globulin ratio. They also need to perform urine analysis (to identify both normal and abnormal constituents). Two competencies of General Medicine, which are integrated with the Biochemistry curriculum and require independent practice, include performing and interpreting capillary blood glucose tests and urine ketone estimation with a dipstick. One competency of Pediatrics, which is integrated with the Biochemistry curriculum and requires independent practice, includes performing and interpreting the urine dipstick test for sugar. 

Integration and Clinical Relevance

The CBME states, “Integration is a learning experience that allows the learner to perceive relationships from blocks of knowledge and develop a unified view of its basis and its application” [13]. Harden [29] discussed 11 steps on the integration ladder, viz., isolation, awareness, harmonization, nesting, temporal, sharing, correlation, complementary, multidisciplinary, interdisciplinary, and transdisciplinary. When we move up on this integration ladder, the significance of an individual discipline diminishes while that of a central curriculum with an organized structure increases. Increased staff participation and joint planning of teaching staff from different disciplines are key to implementing integration [29]. GMER 2018 recommends temporal coordination for integration of teaching [13]. In temporal coordination, the timetable is fixed in coordination with other disciplines. Faculty from different disciplines teach similar topics on the same day or week. Students try to read these topics and gain an integrated idea by linking the topics from the viewpoint of different disciplines [29]. The regulations further highlight that integration in teaching does not always need multiple teachers in the same class. Teachers from different phases and/or subjects may get together and plan a lesson integrating all aspects mentioned in the curriculum. Thereafter, this lesson may be taught by any one of the teachers [13]. This is also known as “nesting” in the integration ladder, where a teacher draws concepts from different subjects in the curriculum to enrich his/her teaching of one subject [29].

**Figure 4:**
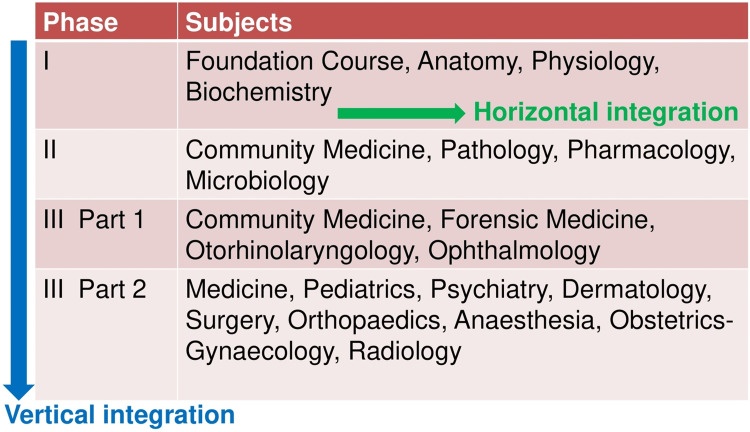
Horizontal and vertical integration in competency-based medical education undergraduate curriculum Images are created by authors using Microsoft PowerPoint (Microsoft® Corp., Redmond, WA).

The two types of integration are mentioned in the CBME undergraduate curriculum: horizontal integration and vertical integration. Horizontal integration connects a topic in different disciplines at the same phase. As per the CBME, eight Physiology competencies are horizontally integrated with the Biochemistry curriculum. Example includes integrating “thyroid hormone synthesis and physiological effects” in Physiology with “thyroid function tests” in Biochemistry. Vertical integration connects a topic in different disciplines of different phases, thereby connecting basic and clinical sciences (Figure 4). 8 Pathology, 4 Dermatology, 1 Ophthalmology, 11 General Medicine, 41 Pediatrics, and 4 General Surgery competencies are vertically integrated with the Biochemistry curriculum. Example includes integrating “source, biochemical functions, and deficiency diseases of vitamin A” in Biochemistry with “vitamin A prophylaxis program in India” in Pediatrics. As per guidelines, not more than 20% of the curriculum of a subject is required to be integrated [13,30].

## Conclusions

Young faculties and senior resident doctors teaching undergraduate medical students need to acquaint themselves with the new CBME curriculum. This review will facilitate those teaching Biochemistry to familiarize themselves with the competency table, learning domains, various teaching methods, and assessment methods. Each student should independently perform the Biochemistry practicals to enhance their skills. Students should be made aware of the competencies they need to acquire as part of their curriculum. Integration with clinical subjects is a key to providing early clinical exposure to students. Integrated teaching helps students gain a blended concept by linking the topics from the viewpoint of different disciplines.
